# Myoferlin regulates epithelial cancer cell plasticity and migration through autocrine TGF-β1 signaling

**DOI:** 10.18632/oncotarget.24971

**Published:** 2018-04-10

**Authors:** Victoria R. Barnhouse, Jessica L. Weist, Vasudha C. Shukla, Samir N. Ghadiali, Douglas A. Kniss, Jennifer L. Leight

**Affiliations:** ^1^ Department of Biomedical Engineering, College of Engineering, The Ohio State University, Columbus, 43210 Ohio, USA; ^2^ The James Comprehensive Cancer Center, The Ohio State University, Columbus, 43210 Ohio, USA; ^3^ Dorothy M. Davis Heart and Lung Research Institute, College of Medicine and Wexner Medical Center, The Ohio State University, Columbus, 43210 Ohio, USA; ^4^ Department of Internal Medicine (Division of Pulmonary, Critical Care and Sleep Medicine), College of Medicine and Wexner Medical Center, The Ohio State University, Columbus, 43210 Ohio, USA; ^5^ Department of Obstetrics and Gynecology (Division of Maternal-Fetal Medicine and Laboratory of Perinatal Research), College of Medicine and Wexner Medical Center, The Ohio State University, Columbus, 43210 Ohio, USA

**Keywords:** myoferlin, TGF-beta, cancer, epithelial-mesenchymal transition, mesenchymal-epithelial transition

## Abstract

Epithelial cancer cells can undergo an epithelial-mesenchymal transition (EMT), a complex genetic program that enables cells to break free from the primary tumor, breach the basement membrane, invade through the stroma and metastasize to distant organs. Myoferlin (MYOF), a protein involved in plasma membrane function and repair, is overexpressed in several invasive cancer cell lines. Depletion of myoferlin in the human breast cancer cell line MDA-MB-231 (MDA-231^MYOFKD^) reduced migration and invasion and caused the cells to revert to an epithelial phenotype. To test if this mesenchymal-epithelial transition was durable, MDA-231^MYOFKD^ cells were treated with TGF-β1, a potent stimulus of EMT. After 48 hr with TGF-β1, MDA-231^MYOFKD^ cells underwent an EMT. TGF-β1 treatment also decreased directional cell motility toward more random migration, similar to the highly invasive control cells. To probe the potential mechanism of MYOF function, we examined TGF-β1 receptor signaling. MDA-MB-231 growth and survival has been previously shown to be regulated by autocrine TGF-β1. We hypothesized that MYOF depletion may result in the dysregulation of TGF-β1 signaling, thwarting EMT. To investigate this hypothesis, we examined production of endogenous TGF-β1 and observed a decrease in TGF-β1 protein secretion and mRNA transcription. To determine if TGF-β1 was required to maintain the mesenchymal phenotype, TGF-β receptor signaling was inhibited with a small molecule inhibitor, resulting in decreased expression of several mesenchymal markers. These results identify a novel pathway in the regulation of autocrine TGF-β signaling and a mechanism by which MYOF regulates cellular phenotype and invasive capacity of human breast cancer cells.

## INTRODUCTION

Metastatic breast cancer is the second leading cause of cancer mortality in women; second only to lung adenocarcinoma, the most lethal of all malignancies [1]. More than 90% of deaths from all cancers are due to spread of tumor cells to distant sites. Mechanistically, epithelial-mesenchymal transition (EMT) is a complex gene program, involving loss of cell-cell and cell-matrix adhesions, elevated levels of matrix metalloproteinase (MMP) activity and enhanced cell mobility and invasion that contributes to the dissemination of cancer cells to distant organs [2, 3]. EMT is characterized by changes in cell morphology and protein expression such as down regulation of E-cadherin, the major epithelial cell-cell adhesion protein, and upregulation of vimentin, the principal mesenchymal intermediate filament protein [3]. Some previous studies have reported that EMT may not be essential for the metastatic process, although cells that exhibit a mesenchymal phenotype have been shown to be more resistant to conventional chemotherapeutic drugs [4, 5].

Myoferlin (MYOF, FER1-L3), a member of the ancient ferlin (FER) family of integral membrane proteins that are important in cell-cell fusion [6], membrane repair [7], endocytosis/exocytosis, and receptor tyrosine kinase (RTK) functions [8–11], has been implicated in the metastatic process. MYOF was previously shown to be important for endocytosis in endothelial cells [12]. MYOF is overexpressed in many epithelial cancers, including invasive mammary and pancreatic adenocarcinomas, oropharyngeal squamous cell carcinoma, and hepatocellular carcinoma [9, 13–15]. Silencing of MYOF in the invasive breast cancer cell line MDA-MB-231 produced a dramatic morphology change from a mesenchymal to an epithelial phenotype. This change was accompanied by changes in protein expression, including upregulation of E-cadherin and downregulation of vimentin and fibronectin, indicating the cells had undergone a mesenchymal to epithelial transition (MET) [13]. Changes in cell behavior were observed as well, with MYOF knockdown cells exhibiting decreased invasion through reconstituted basement membrane and collagen I, and a collective migration pattern, rather than the random migration of the parental cell line [13, 16]. Cells with MYOF knock down also formed smaller, smooth edged tumors that did not invade into surrounding tissue in a mouse xenograft model, with similar results in a study on Lewis lung carcinoma [16, 17].

MYOF has also been shown to be important for endocytosis in endothelial cells. Disruption of myoferlin reduced the endocytosis of transferrin and cholera toxin-B, indicating its importance in clathrin and lipid raft-mediated endocytosis, respectively [12]. A study of pancreatic cancer determined MYOF to be critical for the exocytosis of VEGF, and staining of VEGF and vesicle markers identified an aggregation of vesicles at the cell membrane following MYOF silencing [9]. Studies in MDA-MB-231 cells found MYOF depletion to impede vesicle trafficking and alter the metabolic activity of the cells [18]. MYOF was also found to be a critical regulator of epidermal growth factor receptor (EGFR) signaling and internalization. Epidermal growth factor (EGF) is a potent stimulus of migration in several breast cancer cell lines [19], however depletion of MYOF in MDA-MB-231 and MDA-MB-468 cells blocked EGF- stimulated migration and EMT and reduced tumor development in a chicken chorioallantoic membrane xenograft model of human breast cancer [8].

The molecular mechanism underlying the MET observed after MYOF depletion remains unknown, as well as if loss of MYOF permanently prevents cells from acquiring a mesenchymal phenotype. Therefore, in the current study we investigate if cells with loss of MYOF remain competent to undergo EMT and how MYOF depletion leads to MET.

## RESULTS

### Reversibility of MYOF-induced MET

**Figure 1:**
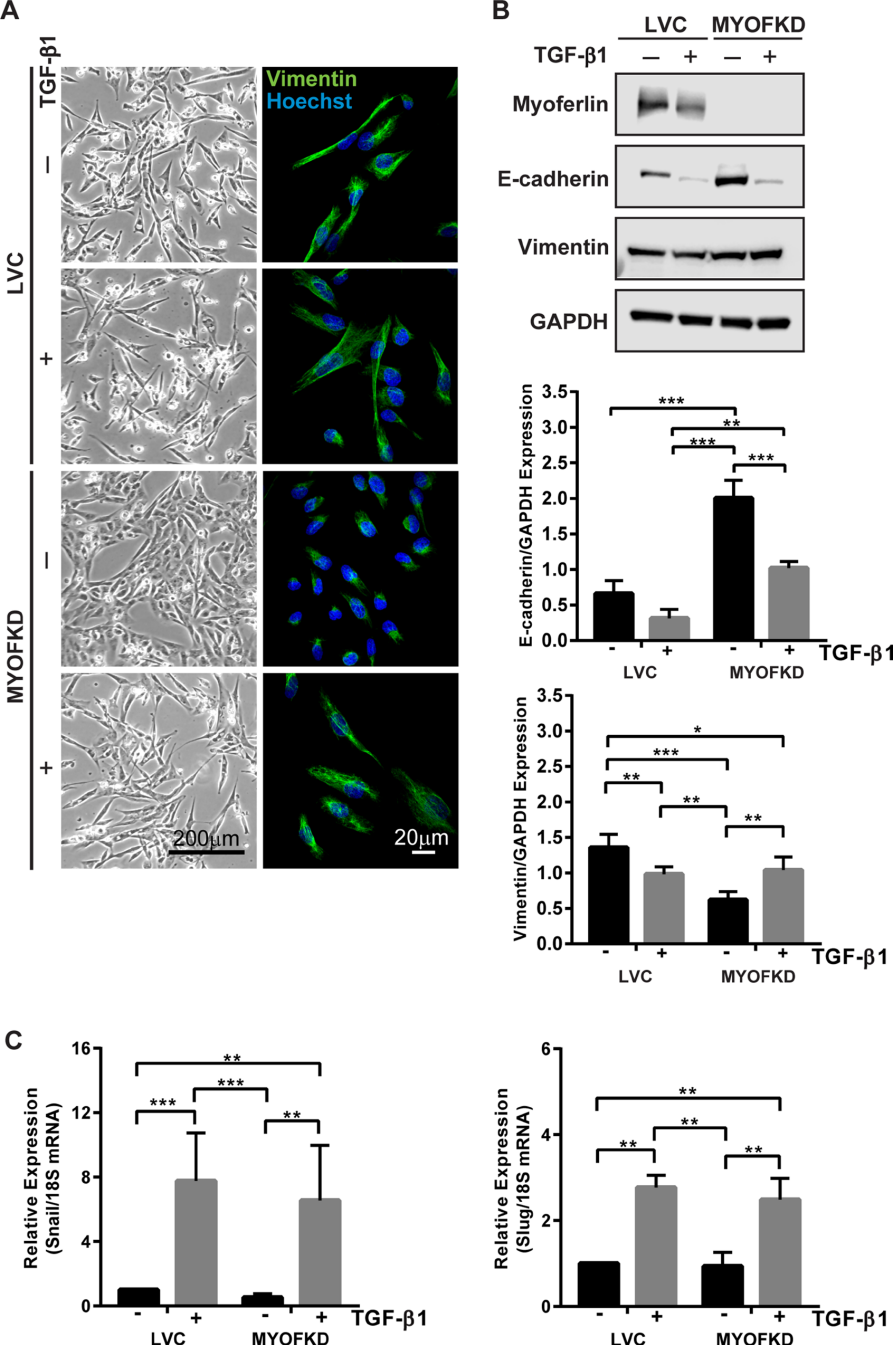
Effect of TGF-β1 treatment on cell phenotype with knockdown of MYOF (MYOFKD) (**A**) Phase contrast and vimentin immunofluorescence images of MDA-231^LVC^ and MDA-231^MYOFKD^ cells after 48 hr TGF-β1 treatment. Phase scale bar = 200 μm, immunofluorescence scale bar = 20 μm. (**B**) Representative images of Western blots of myoferlin, E-cadherin, vimentin, and GAPDH (loading control) and quantification of band intensity normalized to GAPDH. E-cadherin *n* = 3 ± SD; Vimentin *n* = 5 ± SD. (**C**) mRNA expression of Snail and Slug relative to 18S after 2 hr TGF-β1 treatment. *n* = 3 ± SD. ^*^p < 0.05, ^**^p < 0.01, ^***^p < 0.001.

MYOF has been reported to be elevated in invasive cancers and its ablation elicits a change in cell phenotype, yet the mechanism for this is poorly understood. To investigate the role MYOF plays in phenotypic plasticity, we first investigated if loss of MYOF precludes cells from acquiring a mesenchymal phenotype, i.e. undergoing an EMT. MDA-MB-231 cell lines in which MYOF expression was stably knocked down (MDA-231^MYOFKD^ ) were previously generated using lentivirus-based delivery of short hairpin ribonucleic acids (shRNAs) targeting human MYOF, as well as a control cell line (MDA-231^LVC^) using a non-human gene targeting construct [13]. To investigate the plasticity of the cellular phenotype with reduced MYOF, MDA-231^LVC^ and MDA-231^MYOFKD^ were treated with 2 ng/mL transforming growth factor-β1 (TGF-β1), a potent stimulus of EMT. While the morphology of MDA-231^LVC^ was unchanged from the elongated mesenchymal morphology with TGF-β1 treatment, MDA-231^MYOFKD^ cells exhibited a dramatic change from a cobblestone-like epithelial morphology to an elongated morphology, reduced cell-cell adhesion, and more pronounced, organized vimentin intermediate filaments, similar to the MDA-231^LVC^ cellular phenotype (Figure 1A). In addition to the change in morphology characteristic of EMT, corresponding changes in protein expression were also observed, with increased expression of the mesenchymal protein, vimentin, and decreased expression of E-cadherin, the principal epithelial cell-cell adhesion protein, with TGF-β1 treatment of the MDA-231^MYOFKD^ cells (Figure 1B and Supplementary Figures 1, 2). TGF-β1 treatment of the MDA-231^LVC^ cells did not alter cell morphology or E-cadherin protein levels (Figure 1A, 1B). Interestingly, we observed a further decrease in vimentin protein expression with TGF-β1 treatment of the MDA-231^LVC^ cells; however, these levels were similar to the levels of protein expression induced with TGF-β1 in the MDA-231^MYOFKD^ cells.

The TGF-β1-induced EMT was further characterized by mRNA expression of Snail and Slug, transcriptional repressors that inhibit E-cadherin production [20–22]. Both Snail (*Sna1*) and Slug (*Sna2*) were found to be significantly increased in MDA-231^MYOFKD^ cells upon treatment with TGF-β1, further indicating that the MDA-231^MYOFKD^ cells were competent to undergo EMT (Figure 1C). Interestingly, TGF-β1 induced an increase in expression of Snail and Slug to similar levels in both the MDA-231^LVC^ and MDA-231^MYOFKD^ cells, indicating that loss of MYOF does not inhibit the ability of the cells to respond to TGF-β1. Additionally, examination of phosphorylated Smad2, a protein directly activated by TGF-β receptors I and II, by immunoblotting indicated that the MDA-MB-231 cells retained the ability to activate the canonical TGF-β signaling with or without MYOF (Supplementary Figure 2) [23, 24].

In addition to stimulating EMT, TGF-β is a well-established migratory stimulus in MDA-MB-231 cells [25–27]. The effect of MYOF on cell migration has also been in investigated in the MDA-MB-231 cells. Previous studies of MYOF depletion in MDA-MB-231 cells observed changes in migration patterns of the parental cell line, from random, single cell movements in MDA-231^LVC^ to collective migration with an increase in directionality in MDA-231^MYOFKD^ cells due to the increased cell-cell adhesion associated with an epithelial phenotype [16]. However, it was unknown if a TGF-β treatment would affect the migration patterns of the MYOFKD cells in addition to inducing an EMT, particularly as previous studies have found an insensitivity to EGF stimulated migration with MYOF KD [8].

**Figure 2:**
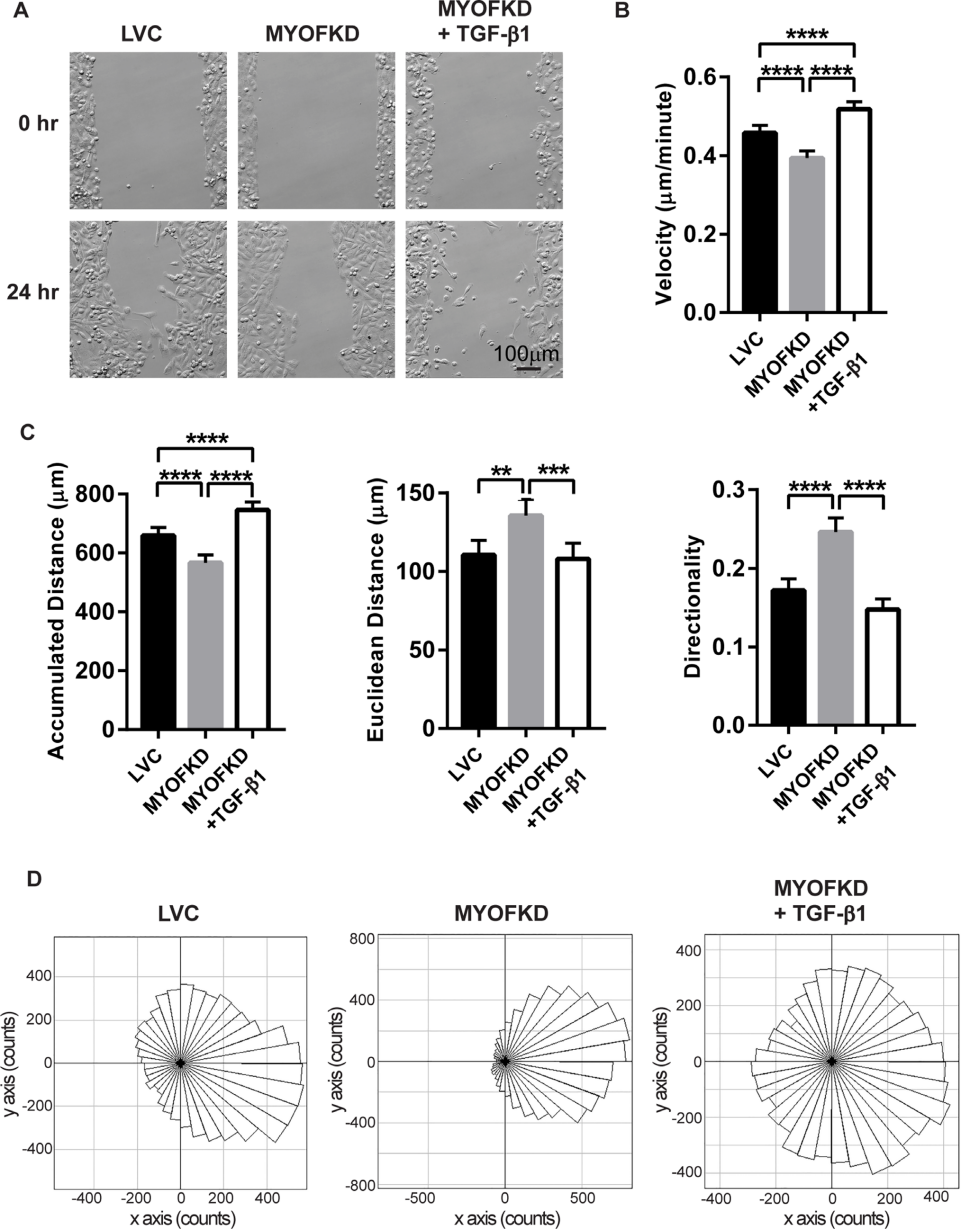
Effect of TGF-β1 treatment on cell migration (**A**) Phase contrast images of MDA-MB-231 cells at 0 hr and 24 hr. Scale bar = 100 μm (**B**) Average velocity of cells in three independent experiments. *n* = 180 ± 95% CI, ^****^p < 0.0001. (**C**) Average accumulated distance, Euclidean distance, and directionality of each cell in three independent experiments. *n* = 180 ± 95% CI, ^**^p < 0.01, ^***^p < 0.001, ^****^p < 0.0001. (**D**) Rose plots representing the directional migration of cell tracks, grouped in 10 degree intervals. *n* = 180 cells per plot.

To examine how TGF-β treatment of MYOF deficient cells affected migration patterns, MDA-231 cells were seeded into a silicone cell culture insert with a defined 500 µm cell-free gap. Cells were treated with TGF-β1 for 48 hr, and then the insert was removed and migration into the gap was followed with time-lapse microscopy for 24 hr (Figure 2A). Images were analyzed to calculate the accumulated distance, Euclidean distance, velocity, and directionality for each cell (Figure 2B, 2C). Similar to previous studies, loss of MYOF resulted in a decrease in velocity and accumulated distance as compared to the LVC cells (Figure 2B). MYOFKD cells also displayed increased collective directed migration, as observed in the phase images at 24 hr with the lack of individual cells, increased Euclidean distance and directionality. Directionality is defined as the straight line distance from the cells’ initial position to final position (Euclidean distance), divided by the total accumulated distance of the cells. A directionality value near one suggests highly directional movement indicative of collective migration, and a value near zero suggests random movement and increased single cell migration. Treatment of MDA-231^MYOFKD^ with TGF-β1 abrogated the trends observed with MYOF depletion, resulting in migration trends similar to MDA-231^LVC^, including increased accumulated distance and velocity (Figure 2B, 2C). Notably, TGF-β1 treatment resulted in a significant decrease in the Euclidean distance and directionality in the TGF-β1 treated MDA-231^MYOFKD^ cells to a value similar to the control MDA-231^LVC^ (Figure 2C). This change in directionality and migration pattern is further illustrated in Rose plots of the cell migration tracks. Cell migration tracks were grouped in 10 degree intervals and movement into the gap was set as a positive change along the x-axis (Figure 2D). MDA-231^MYOFKD^ cells migrate consistently in one direction towards the gap, while TGF-β1 treatment stimulated migration in all directions. This decrease in directionality value indicates a change from the collective migration of the MDA-231^MYOFKD^ cells to single cell migration, which is further supported by the images of TGF-β1 treated MDA-231^MYOFKD^ where single cells migrate away from neighboring cells, much like the MDA-231^LVC^ images (Figure 2A). Untreated MDA-231^MYOFKD^ cells appear as a single cell front, moving together uniformly. The differences in migration pattern were not due to changes in cell proliferation, as DNA content and metabolic activity were unchanged between cell lines or with TGF-β1 treatment (Supplementary Figure 3).

### Knockdown of MYOF reduces autocrine TGF-β production

**Figure 3:**
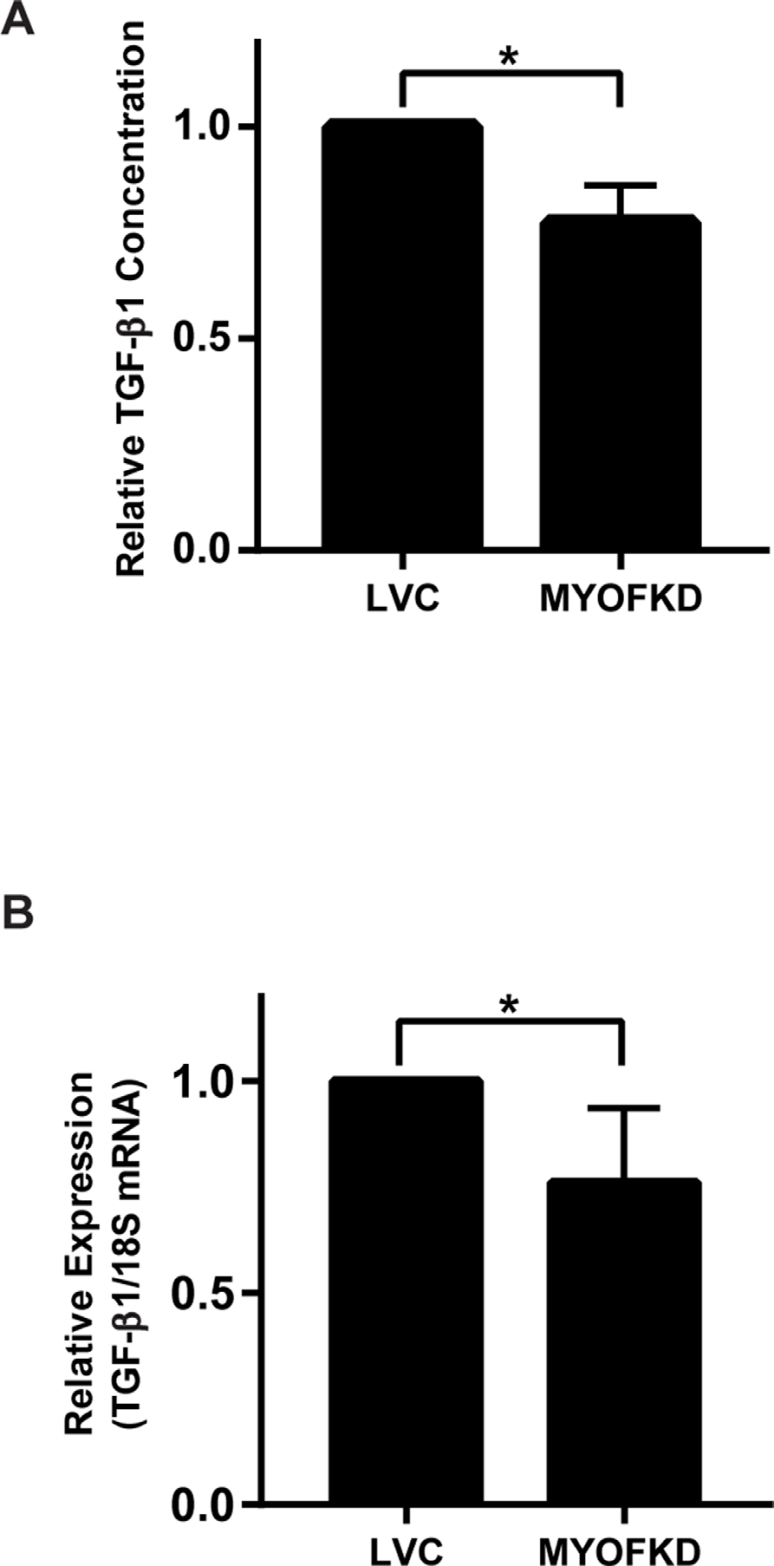
Effect of myoferlin depletion on TGF-β1 expression (**A**) Quantification of TGF-β1 protein concentration in conditioned media by ELISA. *n* = 3 ± SD. (**B**) TGF-β1 mRNA expression relative to 18S determined by quantitative RT-PCR. *n* = 6 ± SD ^*^p < 0.05.

While MDA-231^MYOFKD^ cells remain capable of undergoing EMT, the underlying molecular mechanism of how MYOF regulates cellular phenotype remains unclear. MDA-MB-231 cells produce autocrine TGF-β1 which is required for growth and survival [28], and TGF-β is required for the maintenance of the mesenchymal phenotype [29]. Additionally, MYOF has been previously shown to regulate growth factor secretion, specifically the exocytosis of vascular endothelial growth factor (VEGF) in endothelial cells [9]. Therefore, we hypothesized that MYOF may regulate EMT through autocrine TGF-β production. To determine if TGF-β1 secretion was affected by the loss of MYOF, a TGF-β1 ELISA was used to determine the relative amount of TGF-β1 released into the media of MDA-231^LVC^ and MDA-231^MYOFKD^ cells cultured for 24 hr. MDA-231^MYOFKD^ cells secreted 23% less TGF-β1 than control cells, with an average relative value of 0.77 ± 0.05 (mean ± SD) when normalized to control cells (Figure 3A). While we observed a consistent relative decrease in TGF-β1 concentration in the MDA-231^MYOFKD^ as compared to MDA-231^LVC^, the total concentration of TGF-β1 in conditioned media varied from ∼160 to 500 pg/mL with an average value of 260 pg/mL (Supplementary Figure 4). This range is similar to previous reports of TGF-β1 secreted by MDA-MB-231 cells which was in the range of 200–250 pg/mL [30]. Secretion of TGF-β1 could be regulated by MYOF through several mechanisms, including exocytosis and/or altered gene expression. To determine if gene expression was affected by MYOF depletion, TGF-β1 mRNA expression was analyzed using quantitative RT-PCR. TGF-β1 mRNA expression was significantly decreased by 24% in the MDA-231^MYOFKD^ cells, with an average value of 0.76 ± 0.07 (mean ± SD) when normalized to the control cells (Figure 3B). Because TGF-β1 mRNA expression was reduced by an almost equivalent amount as that observed for the reduction in TGF-β1 secretion (24% vs 23%, respectively), we concluded that changes in mRNA expression were likely responsible for the observed changes in protein expression and did not investigate the effect of MYOF on TGF-β exocytosis.

**Figure 4:**
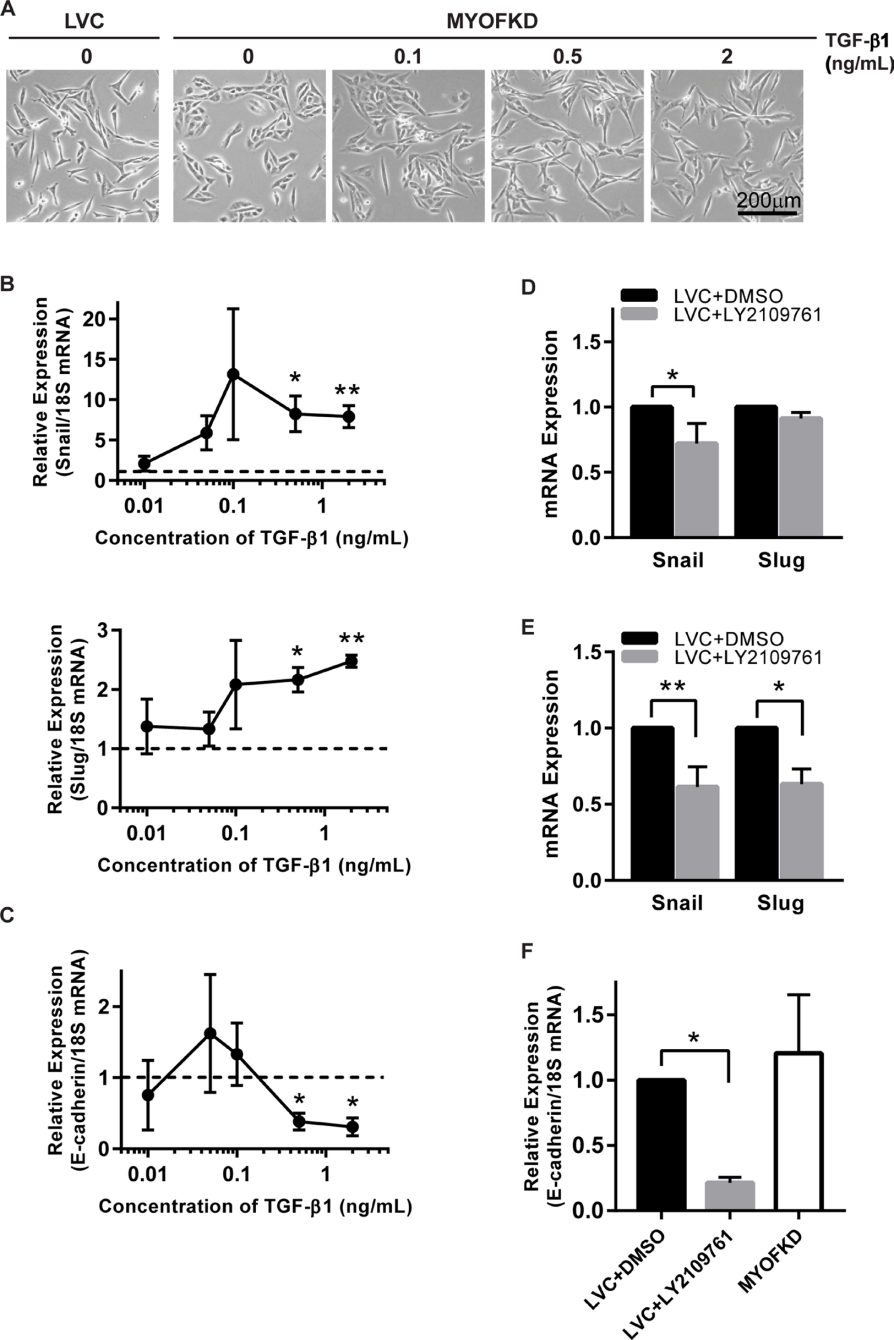
Sensitivity of cell phenotype to TGF-β signaling (**A**) Phase contrast images of MDA-231^MYOFKD^ cells after 24 hr of treatment with TGF-β1. Scale bar = 200 μm. (**B**) Expression of Snail and Slug mRNA relative to 18S after 2 hr TGF-β1 treatment. Dotted lines indicates expression of MDA-231^MYOFKD^ BSA control. Snail: *n* = 4, Slug: *n* = 3 ± SD ^*^p < 0.05, ^**^p < 0.01, all conditions compared with MDA-231^MYOFKD^ + BSA control. (**C**) Expression of E-cadherin mRNA relative to 18S after 24 hr TGF-β1 treatment. *n* = 3 ± SD ^*^p < 0.05, all conditions compared with MDA-231^MYOFKD^ BSA control. (**D**) Expression of Snail and Slug after 2 hr LY2109761 treatment, relative to LVC DMSO control. Snail: *n* = 6 ± SD, Slug: *n* = 3 ± SD ^*^p < 0.05. (**E**) Expression of Snail and Slug after 24 hr LY2109761 treatment, relative to LVC DMSO control. Snail: *n* = 6 ± SD, Slug: *n* = 3 ± SD ^*^p < 0.05, ^**^p < 0.01. (**F**) Expression of E-cadherin mRNA relative to 18S after 24 hr LY2109761 treatment. *n* = 3 ± SD ^*^p < 0.05.

While TGF-β1 is a potent growth factor, it was not clear if a 20% decrease in concentration was sufficient to induce EMT. To determine the concentration of TGF-β1 necessary to induce an EMT, MDA-231^MYOFKD^ cells were treated with a range of TGF-β1 concentrations from 0.01 to 2 ng/mL. At short time points (2 hr), as little as 0.1 ng/mL of TGF-β1 resulted in an increase in Snail and Slug expression (Figure 4B). Expression of ZEB1, an additional mesenchymal transcription factor associated with EMT, was also quantified, however ZEB1 expression was unchanged for all concentrations of TGF-β1 investigated (Supplementary Figure 5A). After 24 hr of TGF-β1 treatment, E-cadherin mRNA expression was significantly decreased with concentrations of 0.5 ng/ml of TGF-β1 or greater (Figure 4C). Distinct changes in morphology to a more elongated shape were observed with prolonged exposure of 0.5 ng/mL of TGF-β1 (Figure 4A). These values fall within the range of TGF-β1 production observed with the ELISA assays, and suggest that even small changes in the concentration of TGF-β1 can affect cellular phenotype.

### Inhibition of TGF-β1 receptor signaling reduces Snail and Slug expression

While TGF-β1 is a potent inducer of EMT, and we observed significant decreases in TGF-β1 secretion in the MDA-231^MYOFKD^ cells, it remained unclear if the reduction of autocrine TGF-β1 alone was sufficient to cause an MET in the MDA-MB-231 control cell line. To test this, TGF-β signaling was blocked by treating MDA-231^LVC^ cells with the pharmacological inhibitor LY2109761, which targets TGF-β type I and II receptor kinases. MDA-MB-231 cells require a basal level of autocrine TGF-β1 signaling for survival, and complete inhibition of this pathway has been shown to lead to high cell death rates [28]. Therefore, a short term (<24 hr) inhibition model was used rather than a total knockdown of TGF-β1 production. Inhibition of the TGF-β signaling pathway with 2 μM LY2109761 for 2 hr decreased Snail expression in the MDA-231^MYOFKD^ as compared to the MDA-231^LVC^ (Figure 4D). This effect was more pronounced at 24 hr, with a significant decrease in both Snail and Slug expression (Figure 4E). No changes were observed in expression of ZEB1 (Supplementary Figure 5B, 5C). Interestingly, E-cadherin expression was reduced with 24 hr LY2109761 treatment of the MDA-231^LVC^ (Figure 4F); therefore inhibition of TGF-β signaling alone may not be sufficient to rescue E-cadherin expression. However, no changes were observed in mRNA expression of E-cadherin between the MDA-231^LVC^ and MDA-231^MYOFKD^, in contrast to the increased E-cadherin protein expression observed with loss of MYOF (Figure 1B), similar to previous reports [13]. Therefore, we hypothesize that E-cadherin protein expression is likely not transcriptionally regulated at this time point. Previous reports have shown that autocrine TGF-β signaling is necessary for survival in MDA-MB-231 cells [28], and we observe similar toxic effects with prolonged inhibition (48–72 hr) of TGF-β signaling by the LY2109761 compound, including the formation of apoptotic bodies and large vacuoles (Supplementary Figure 6). Therefore, we were not able to investigate the effects of inhibiting TGF-β signaling on protein expression associated with EMT, which would require longer time points (48–72 hr).

## DISCUSSION

MYOF is overexpressed in multiple types of cancer including breast, pancreatic, oropharyngeal squamous cell, and hepatocellular carcinomas. Our group demonstrated that knockdown of MYOF in MDA-MB-231 cell results in a MET (i.e., reverted to epithelial morphology) and dramatically impaired motility and invasion [13, 16]. In this work, we examined whether MYOF-depleted MDA-MB-231 breast cancer cells would exhibit altered TGF-β receptor signaling and be refractory to undergoing an EMT in response to the growth factor. In fact, we observed that the cells were able to transition to mesenchymal cells both genetically and morphologically when incubated with low concentrations of TGF-β.

The principal finding in the current work demonstrated that, although depletion of MYOF in highly invasive MDA-MB-231 cells (i.e., MDA-231^MYOFKD^) caused a morphologically and genetically identifiable MET that reduced motility and invasion, this phenotype was not immutable. That is, the canonical EMT inducer TGF-β overcame MYOF silencing and still elicited an EMT. This suggests that the epithelial and mesenchymal genetic phenotypes are, at least somewhat, interconvertible. Moreover, we recently reported that MYOF depletion resulted in activation of focal adhesion kinase (FAK), activation of mature focal adhesions and enhanced cell-matrix adhesion [31]. Recent work by Zhang *et al.* [32] indicates that the EMT is bistable with a “partial EMT” involving an intermediate gene expression program, and therefore a metastate between a fully epithelial phenotype and one that is consistent with a mesenchymal cell. In their model, TGF-β induced early gene expression of Snail leading to down-regulation of E-cadherin. This elicits a metastable or partial EMT state that can be reversed to an epithelial phenotype or further consolidated to a fully mesenchymal morphology by induction of ZEB1. This latter phenotype can be either reversible or stable depending upon the duration and concentration of TGF-β [32].

We suggest that MYOF may be part of that transition, and this opens enormous opportunities to explore this pathway. In addition, given that MYOF is overexpressed in multiple types of epithelial cancers, including breast adenocarcinoma [17], ductal pancreatic adenocarcinoma [33], non-small cell lung adenocarcinoma [17, 34], squamous cell carcinoma [14], hepatocellular carcinoma [11], and melanoma [35], we suggest that MYOF will be a substantial participant in the transition from a stable parenchymal cell in epithelia to a premetastatic and then completely metastatic cell that invades and disseminates to distant organs to form secondary tumors.

In addition, we noted that TGF-β receptor signaling remained essentially intact, as cells responded to the growth factor by up-regulation of the transcriptional repressor Snail and Slug. This is in contrast to a previous study investigating the response of MDA-MB-231 cells to epidermal growth factor (EGF) stimulation [8], in which MYOF depletion ablated EGF stimulated migration and EMT. Our results further suggest a role for autocrine TGF-β signaling as one underlying mechanism of MET after MYOF depletion.

## MATERIALS AND METHODS

All reagents, cell lines, commercial kits, software packages, and primers are listed in Table 1 with catalog numbers (if appropriate) and vendor source.

**Table 1:** Key Reagents and Resources

Reagent or Resource	Source	Identifier
**Antibodies**		
Mouse Anti-E-cadherin, Clone 36	BD Transduction Laboratories, San Jose, CA, USA	Cat#610181; RRID: AB_397580
Mouse Anti-Vimentin, Clone V9	Millipore, Burlington, MA, USA	Cat#MAB3400; RRID: AB_94843
Mouse Anti-GAPDH Monoclonal, Unconjugated, Clone 6C5	Novus Biologicals, Saint Charles, MO, USA	Cat#NB600-502; RRID: AB_350715
Rabbit Anti-Myoferlin, Polyclonal	Sigma-Aldrich, St. Louis, MO, USA	Cat#HPA014245; RRID: AB_1848495
Mouse Anti-β-Actin Monoclonal, Clone AC-15	Sigma-Aldrich, St. Louis, MO, USA	Cat# A1978-100UL
Rabbit Anti-Phospho-Smad2 Monoclonal, Clone 138D4	Cell Signaling Technology	Cat# 3108S; RRID: AB_490941
**Chemicals, Peptides, and Recombinant proteins**		
Recombinant Human TGF-β1	PeproTech, Rocky Hill, NJ, USA	Cat# 100-21
**Critical commercial assays**		
Human TGF-β1 Quantikine ELISA kit	R&D Systems, Minneapolis, MN, USA	Cat# DB100B
Sample Activation Kit 1	R&D Systems	DY010
RNeasy Mini Kit	Qiagen, Hilden, Germany	Cat# 74104
CyQuant Cell proliferation assay	Life Technologies, Carlsbad, CA, USA	Cat# C7026
**Experimental models: Cell lines**		
MDA-MB-231 LVC	Laboratory of Douglas Kniss, [13] Li *et al.* 2012	N/A
MDA-MB-231 MYOF-KD	Laboratory of Douglas Kniss, [13] Li *et al.* 2012	N/A
**Primers**		
Human TGF-β1 Forward: GGCGATACCTCAGCAACCG	[37] Yin *et al.*, 2016	
Human TGF-β Reverse: AAGGCGAAAGCCCTCAAT	[37] Yin *et al.*, 2016	
Human Snail Forward: ACCACTATGCCGCGCTCTT	[38] Medici *et al.*, 2008	
Human Snail Reverse: GGTCGTAGGGCTGCTGGAA	[38] Medici *et al.*, 2008	
Human Slug Forward: AGATGCATATTCGGACCCAC	[39] Dhasarathy *et al.*, 2007	
Human Slug Reverse: CCTCATGTTTGTGCAGGAGA	[39] Dhasarathy *et al.*, 2007	
Human E-Cadherin Forward: TGCTCTTGCTGTTTCTTCGG	[40] Yang *et al.*, 2017	
Human E-Cadherin Reverse: TGCCCCATTCGTTCAAGTAG	[40] Yang *et al.*, 2017	
Human Vimentin Forward: GAAGAGAACTTTGCCGTTGAAG	[40] Yang *et al.*, 2017	
Human Vimentin Reverse: GAGAAATCCTGCTCTCCTCG	[40] Yang *et al.*, 2017	
Human ZEB1 Forward: CCTAGATCAGGACTCAAGAC	[41] Eger *et al.*, 2005	
Human ZEB1 Reverse: CACAGAAGGCAAGTGCTATC	[41] Eger *et al.*, 2005	
18S	Life Technologies	Cat# AM1716
**Software and Algorithms**		
GraphPad Prism, Ver 7.00	GraphPad Software, La Jolla, CA, USA	N/A
Image Studio Lite, Ver 5.2	LI-COR, Lincoln, NE, USA	N/A
ImageJ, Ver 1.43u	NIH, Bethesda, MD, USA	N/A
**Other**		
Silicon wound assay chambers	Ibidi, Fitchburg, WI, USA	Cat#80209

### Cell culture

MDA-MB-231 breast cancer cells with a stable lenti-viral knockdown of MYOF (MDA-231^MYOFKD^) and lenti-viral control cells (MDA-231^LVC^) were maintained in Dulbecco’s modified Eagle’s medium (DMEM, Gibco, Carlsbad, CA, USA) with 4.5 g/L D-glucose supplemented with 10% fetal bovine serum (FBS, Seradigm, Radnor, PA, USA), 1% L-glutamine (Gibco) and 1% penicillin-streptomyosin (Gibco) [13]. Identity of cell lines was verified by short tandem repeat (STR) profiling through ATCC (Manassas, VA, USA). Cells were seeded at a density of 2 × 10^4^ cells/cm^2^ in normal growth media overnight before treatment for all experiments unless otherwise noted.

### Immunofluorescence staining and imaging

Cells were plated on 12 mm glass coverslips overnight, then treated with 2 ng/mL TGF-β1 or the vehicle control (4 mM HCL 0.1% bovine serum albumin (BSA)) for 48 hr. Cells were fixed with 4% paraformaldehyde for 10 min, permeabilized with 0.5% Triton X-100 (Sigma-Aldrich, St. Louis, MO, USA) for 5 min, and blocked for 1 hr with 10% normal goat serum at room temperature (RT). Samples were incubated with primary antibodies (1:500) over night at 4° C, rinsed with PBS for 10 minutes, 3 times, and incubated with Alexa Fluor 488 (1:1000) and Hoechst (1:1000) for 1 hr at RT, then rinsed with PBS for 10 minutes, 3 times. Coverslips were mounted onto glass slides with ProLong Gold Antifade Mountant (Life Technologies, Carlsbad, CA, USA), and imaged at RT using an inverted confocal microscope (Olympus FV3000; Olympus, Tokyo, Japan) equipped with a 40×, UPlanFl N, NA 1.3, oil immersion objective. Phase images were captured with an Evos XL Core (AMEX1200, Life Technologies).

### TGF-β1 enzyme linked immunosorbent assay (ELISA)

Cells were plated overnight, rinsed with PBS, and cultured for 48 hr in serum free growth media. Culture media was collected and assayed immediately according to the human TGF-β1 Quantikine ELISA kit (R&D Systems, Minneapolis, MN, USA) manufacturer’s protocol. Samples were activated prior to running the assay using the Sample Activation Kit 1 (R&D Systems). Absorbance values were measured at 450 nm and 540 nm with a Spectra Max M2 plate reader (Molecular Devices, Sunnyvale, CA, USA). The 540 nm values were subtracted from the 450 nm readings in order to correct for optical imperfections within each plate, as per the manufacturer’s protocol.

### Quantitative RT-PCR

Prior to treatment, cells were starved for 2 hr in serum free growth media. Cells were then treated with 2, 0.5, 0.1, 0.05, or 0.01 ng/mL TGF-β1 or BSA vehicle control for 2 hr. For inhibitor assays, LY2109761 (Selleck Chemicals, Houston, TX, USA), a pharmacological inhibitor of TGF-β receptor type I/II, was dissolved in DMSO at a concentration of 2 mM. Cells were treated with 2 µM LY2109761 or DMSO vehicle control. Cells were lysed and RNA isolation was performed using the RNeasy Mini Kit (Qiagen, Hilden, Germany) according to the manufacturer’s protocol, homogenized using Qiashredder columns (Qiagen), and on column DNAse digestion. Quantification of total RNA was performed using a NanoDrop 2000 (Thermo Fisher Scientific, Waltham, MA, USA). Total RNA (0.5 µg) was reverse transcribed to cDNA using iScript master mix (Bio-Rad, Hercules, CA, USA). Equal amounts of cDNA per condition, SYBR green master mix (Applied Biosystems, Foster City, CA, USA), and primer probe sets (see methods Table) were detected with a StepOnePlus RT- PCR system (Applied Biosystems). The mRNA fold changes were determined using the ΔΔCT method normalized to the 18S endogenous control [36].

### Protein extraction and immunoblotting

Cells were cultured for 48 hr in serum free growth media supplemented with Insulin-Transferrin-Selenium (Life Technologies), and 2 ng/mL TGF-β1 or BSA vehicle control. Cells were rinsed with ice-cold PBS and lysed for 20 minutes on ice using RIPA buffer (Life Technologies) and 1:100 dilution of Halt protease and phosphatase inhibitor cocktail (Thermo Fisher Scientific). Samples were centrifuged at 12,000 rpm for 15 minutes at 4° C and the supernatant was transferred to fresh pre-chilled tubes. Protein concentrations were determined using a μBCA protein assay (Thermo Fisher Scientific). Equal amounts of total protein (30 μg) were loaded onto a 4–12% Bis-Tris Plus Gel (Life Technologies), separated via SDS-PAGE, and then transferred to a PVDF membrane (Thermo Fisher Scientific). Membranes were blocked with 5% BSA (Sigma-Aldrich) or 5% milk (Lab Scientific, Inc, Highlands, NJ, USA) in Tris-buffered saline with 0.05% Tween20 (TBS-T, Sigma-Aldrich) for 1 hr at room temperature and then probed with the following primary antibodies diluted in BSA blocking solution: E-cadherin (1:2000, BD Biosciences, San Jose, CA, USA), vimentin (1:1000, Millipore, Burlington, MA, USA), phospho-Smad2 (1:1000, Cell Signaling Technologies), β-actin (1:4000, Sigma Aldrich) myoferlin (1:1000, Sigma-Aldrich), and glyceraldehyde 3-phosphate dehydrogenase (GAPDH, 1:2000, Novus Biologicals, Littleton, CO, USA). Myoferlin (1:1000, Sigma-Aldrich) was diluted in 5% milk. Membranes were incubated with primary antibodies overnight in 4° C, rocking constantly. Membrane sections were then rinsed with TBS-T, 3 times for 10 minutes each, then incubated with horseradish peroxidase-conjugated secondary antibodies (1:1000, Thermo Fisher Scientific) for 1 hr at RT, and then rinsed again with TBS-T, 3 times for 10 minutes each. Chemiluminescent substrate SuperSignal West femto (Thermo Fisher Scientific) for E-cadherin, and SuperSignal West pico (Thermo Fisher Scientific) for vimentin, myoferlin and GAPDH, were added to the appropriate membrane sections to detect protein bands. Membranes were visualized using the Odyssey Fc Imaging System (LI-COR, Lincoln, NE, USA). Protein bands were visualized using the chemiluminescent channel, Precision Plus Protein Dual Color Standard ladder (Bio-Rad) was visualized using the 700 nm channel. Bands were quantified using ImageJ (NIH, Bethesda, MD, USA).

### Cell migration

Migration studies were performed using 2-well silicone wound assay inserts (Ibidi, Fitchburg, WI, USA) which can be removed to create a gap between defined cell edges without damaging the cell monolayer that has formed. MDA-MB-231 cells were seeded into each insert with normal growth media, and incubated overnight. MDA-231^LVC^ cells were treated with the BSA vehicle control and MDA-231^MYOFKD^ cells were treated with 2 ng/mL TGF-β1 or vehicle control in basal growth media supplemented with 10% charcoal stripped FBS for 48 hr. The Ibidi inserts were then carefully removed and the cells washed with PBS to remove any non-adherent cells. Fresh media with TGF-β1 or vehicle control was added. Cells were then imaged using an inverted fluorescence microscope (Olympus IX81) with live cell imaging chamber (Okolab, Burlingame, CA, USA) to record bright field images every ten minutes over 24 hr. Time-lapse images were analyzed using ImageJ (NIH) to track individual cell migration. Cell position data were then analyzed using MATLAB to determine accumulated distance, cell velocity, Euclidean distance, and cell directionality. The mean cell directionality was found according to:

$$Directionality=\frac{1}{n}\sum_{i=1}^{n}\frac{d_{acc}}{d_{Euc}}$$

where *d*_*Euc*_ is the distance between the initial and final positions of each cell, *d*_*acc*_ is the accumulated distance between each time point for each cell, and *n* is the total number of cells tracked per experiment.

### Proliferation assay

Cells were incubated in serum free growth media supplemented with Insulin-Transferrin-Selenium (Life Technologies) plus 2 ng/mL TGF-β1 or vehicle control and incubated for 48 hr. Plates were then rinsed with PBS and frozen at –70° C for at least 24 hr prior to assay. A CyQuant Cell Proliferation Assay kit (Life Technologies) was then used to detect total amounts of DNA content per condition according to the manufacturer’s protocol.

### Metabolic activity assay

Cells were incubated in serum free growth media supplemented with Insulin-Transferrin-Selenium (Life Technologies) plus 2 ng/mL TGF-β1 or vehicle control and incubated for 48 hours. AlamarBlue (Life Technologies) was added directly to the cells to create a 1:10 final dilution, and incubated for 6 hr. Fluorescence was measured with a Synergy HT plate reader (BioTek, Winooski, VT, USA) at 560 nm excitation/590 nm emission.

### Statistical analysis

Data analysis for graphical representation and statistical results were performed using GraphPad Prism version 7.00 (GraphPad). Error bars represent the standard deviation of the samples, unless otherwise noted in the figure caption. For data sets with 3 or more groups and two changing factors, statistical analysis was performed using a two-way ANOVA followed by a Tukey’s Multiple Comparison Test with an alpha level of 0.05. Data sets with three or more groups and one changing factor, a one-way ANOVA followed by a Dunnett’s test was used with an alpha of 0.05. For data with two sets that were normally distributed, an unpaired one-tailed *t*-test was conducted to determine significance, with an alpha level of 0.05.

## SUPPLEMENTARY MATERIALS FIGURES



## Abbreviations

EMT: epithelial-mesenchymal transition
LVC: lentiviral control
MET: mesenchymal-epithelial transition
MYOF: myoferlin
MYOFKD: myoferlin knockdown
STR: short tandem repeats
TGF-β: transforming growth factor-β
