# Supplementary material for: Myoferlin regulates epithelial cancer cell plasticity and migration through autocrine TGF-β1 signaling
Source: Oncotarget. 2018 Apr 10;9(27):19209–22. doi: 10.18632/oncotarget.24971 (PMC5922389; doi:10.18632/oncotarget.24971)
Supplement: Supplementary file 1 [file oncotarget-09-19209-s001.pdf]

Myoferlin regulates epithelial cancer cell plasticity and migration through autocrine TGF-β1 signaling

SUPPLEMENTARY MATERIALS

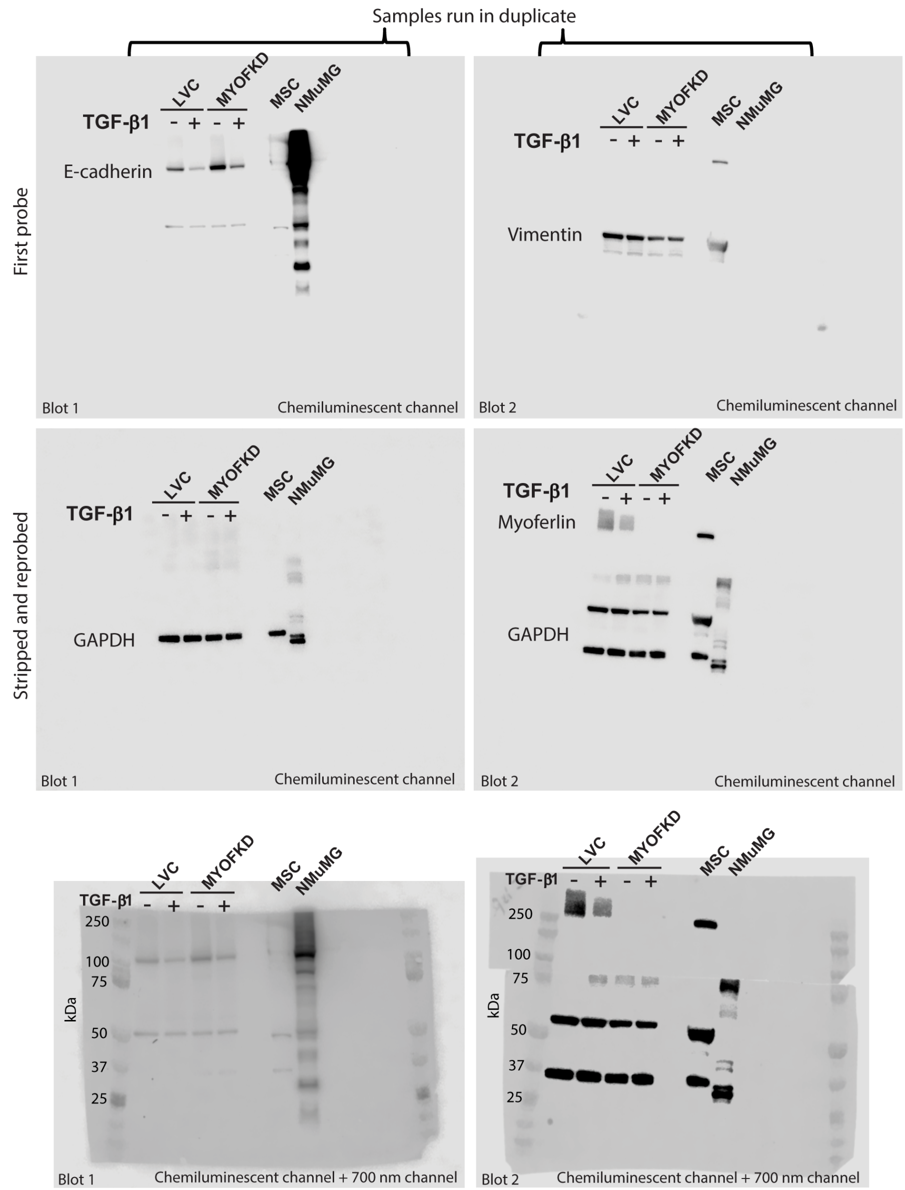

Supplementary Figure 1: Uncropped images of Western blots and controls from Figure 1. Vimentin and MYOF positive control, E-cadherin negative control – human mesenchymal stems cells (MSC). Vimentin and MYOF negative control, E-cadherin positive control – normal murine mammary gland cell line (NMuMG).

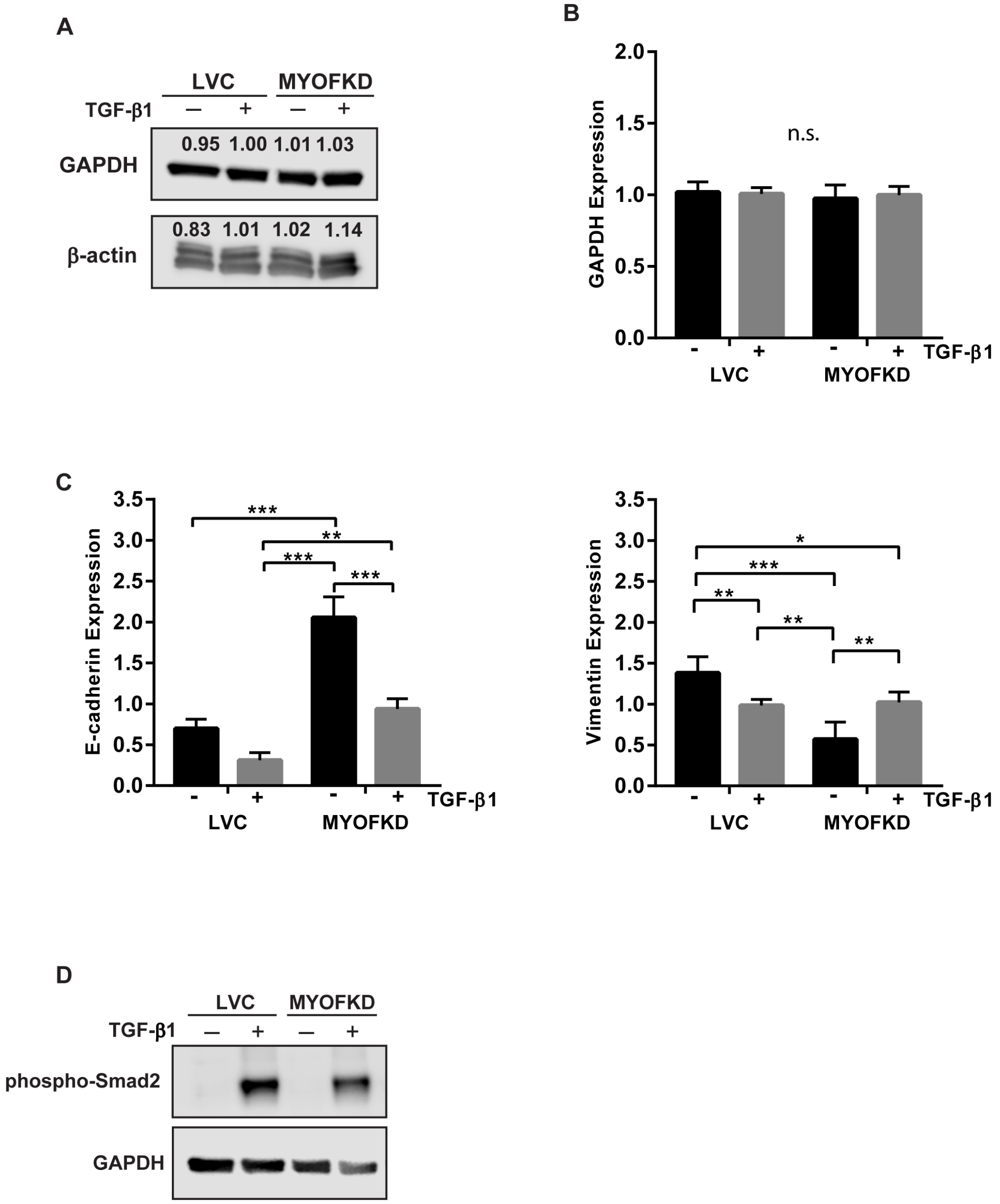

Supplementary Figure 2: (**A**) ImageJ quantification of individual bands from western blot in Figure 1, indicated by number directly above the band. β-actin included as an additional loading control. (**B**) Quantification of all GAPDH bands from experiments in Figure 1. No significant (n.s.) differences observed between conditions, $n = 5 + SD$. (**C**) Quantification of vimentin ($n = 5 + SD$) and E-cadherin ($n = 3 + SD$) from Figure 1 without normalization to GAPDH. $^{*}p < 0.05$, $^{**}p < 0.01$, $^{***}p < 0.001$. (**D**) Western blot for phospho-Smad2 after 1 hr of TGF-β1 treatment.

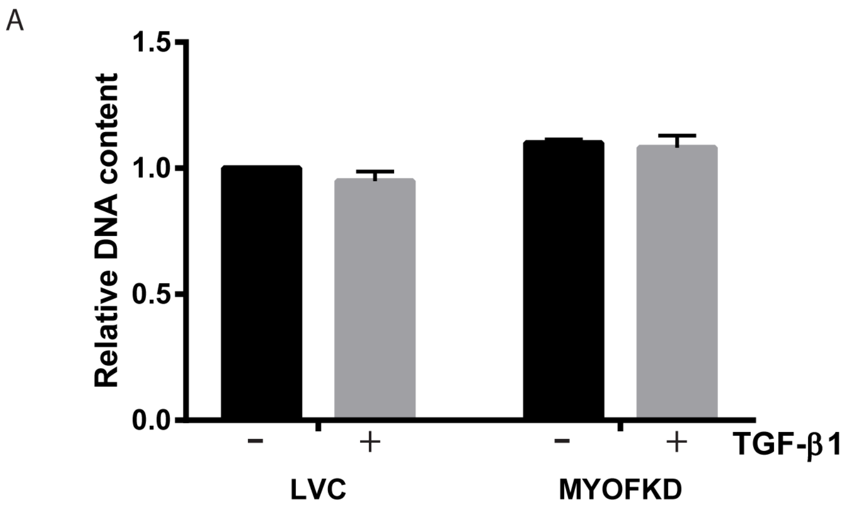

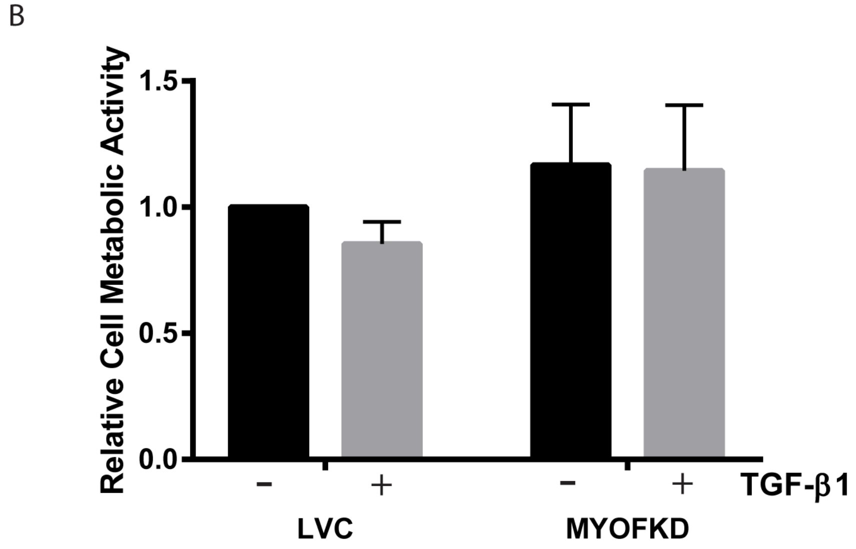

Supplementary Figure 3: Effect of TGF-β1 on cell proliferation after 48 hr TGF-β1 treatment. (**A**) DNA content determined by CyQUANT cell proliferation kit. Values were normalized to LVC control. $n = 3 \pm$ SD n.s. (**B**) Metabolic activity measured with alamarBlue reagent and normalized to LVC control. $n = 3 \pm$ SD n.s.

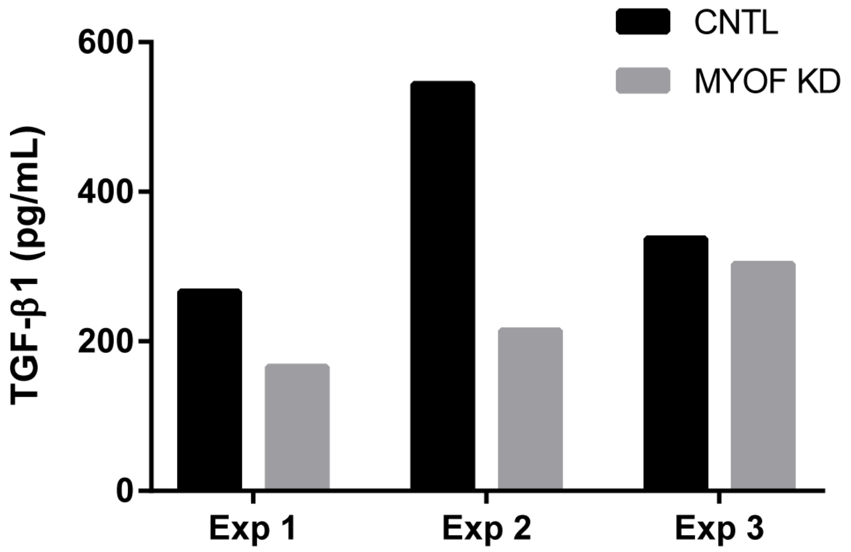

Supplementary Figure 4: Representative experiments of TGF-β1 concentration as determined by ELISA.

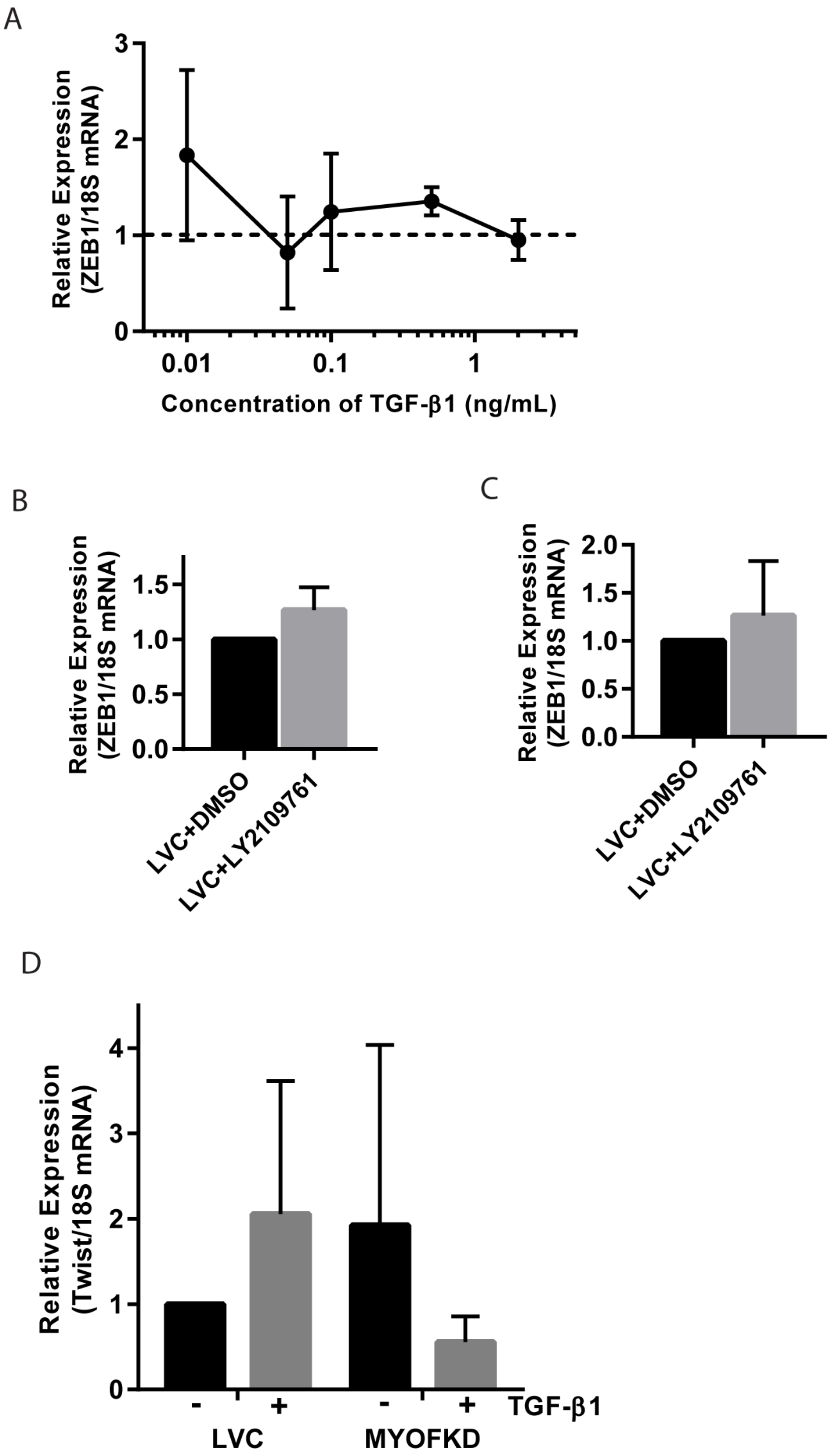

Supplementary Figure 5: (**A**) Effect of TGF-β1 treatment on ZEB1 mRNA expression of MDA-231^{MYOFKD} cells after 2 hr TGF-β1 treatment. $n = 3 \pm$ SD, n.s. (**B**) Expression of ZEB1 mRNA relative to 18 S after 2 hr LY2109761 treatment. $n = 3 \pm$ SD, n.s. (**C**) Expression of ZEB1 mRNA relative to 18 S after 24 hr LY2109761 treatment. $n = 3 \pm$ SD, n.s. (**D**) Expression of TWIST mRNA relative to 18 S after 2 hr TGF- β1 treatment. $n = 3 \pm$ SD, n.s.

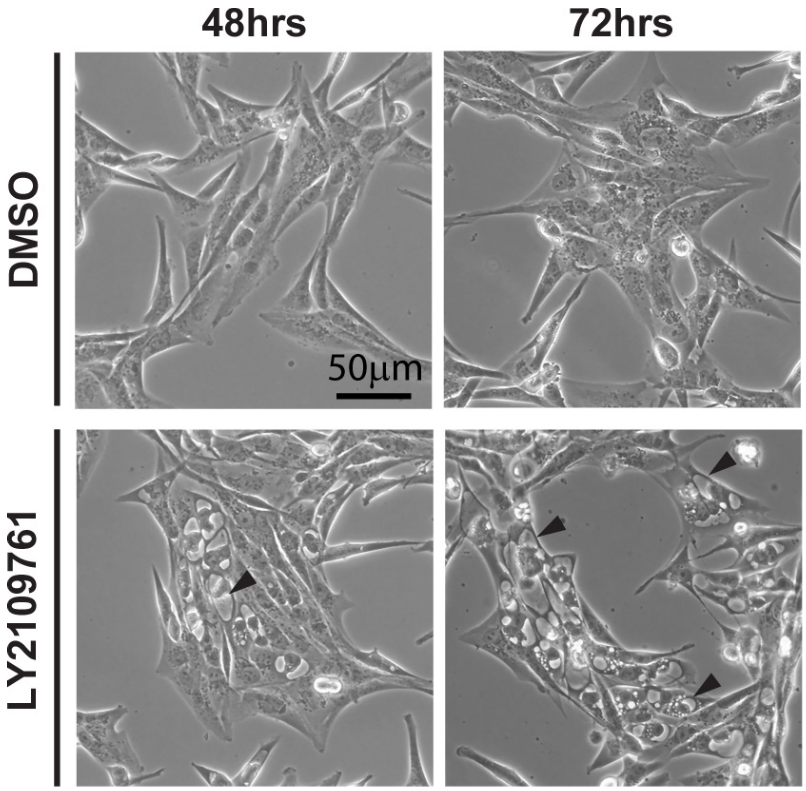

Supplementary Figure 6: Phase contrast images of MDA-231^{LVC} cells after 48 hr and 72 hr of treatment with LY2109761, where the presence of large vacuoles (arrow heads) within the cells can be observed. Scale bar = 50 μm.
